# Phenotypic characterization of neurofibromatosis type 1 in a large Chinese cohort: A cross-sectional study

**DOI:** 10.1016/j.jdin.2025.09.020

**Published:** 2025-10-16

**Authors:** Zhichao Wang, Xing Hu, Haoyang Mo, Minxue Shen, Carlos G. Romo, Katya Vera, Zhixiong Liu, Qingfeng Li, Chuntao Li, Kavita Y. Sarin

**Affiliations:** a Department of Plastic and Reconstructive Surgery, Shanghai Ninth People’s Hospital, Shanghai Jiao Tong University School of Medicine, Shanghai, China; b Neurofibromatosis Type 1 Center and Laboratory for Neurofibromatosis Type 1 Research, Shanghai Ninth People’s Hospital, Shanghai Jiao Tong University School of Medicine, Shanghai, China; c Pazhou Lab, Guangzhou, China; d Department of Dermatology, Hunan Provincial People's Hospital (The First Affiliated Hospital of Hunan Normal University), Changsha, China; e Department of Neurosurgery, Xiangya Hospital, Central South University, Changsha, China; f National Clinical Research Center for Geriatric Disorders, Xiangya Hospital, Central South University, Changsha, China; g Department of Social Medicine and Health Management, Xiangya School of Public Health, Central South University, Changsha, China; h Department of Neurology, Johns Hopkins University School of Medicine, Baltimore, Maryland; i Department of Dermatology, Stanford University School of Medicine, Redwood City, California

**Keywords:** neurofibroma, neurofibromatosis type 1, racial differences, subtypes

## Abstract

**Background:**

Neurofibromatosis type 1 (NF1) is a multisystemic genetic disorder characterized by *NF1* gene mutations. The well-described manifestations of NF1 are primarily derived from European populations.

**Objective:**

To evaluate the phenotypic characteristics of Chinese patients with NF1.

**Methods:**

This cross-sectional, multicenter study involved patients with NF1 from Chinese and U.S. populations. Clinical data were obtained from NF1 clinics and patient-reported outcome assessments.

**Findings:**

1313 patients with Chinese NF1 were included. We found that Chinese patients were diagnosed later in life and exhibited a lower prevalence of spinal neurofibromas and fractures but a higher rate of malignant peripheral nerve sheath tumors as compared to U.S. patients. Four distinct NF1 subtypes were identified: severe neural, cutaneous, hereditary, and mild. Significant correlations were observed between freckles and cutaneous neurofibromas and between bone lesions and pseudarthrosis. Cutaneous manifestations were correlated with systemic manifestations, and the risk of developing plexiform neurofibromas was inversely proportional to the number of cutaneous neurofibromas and freckles.

**Limitations:**

The use of self-report data may introduce subjectivity and recall bias.

**Conclusion:**

This study provides the first comprehensive analysis of clinical phenotypes in Chinese patients with NF1. The identification of distinct NF1 subtypes, phenotypic correlations, and ethnic differences supports more personalized management strategies for NF1 patients.

**Capsule Summary**

• Studies on clinical features of Chinese patients with neurofibromatosis type 1 (NF1) are lacking.
• This study provides a comprehensive overview of the clinical phenotypes within this population, demonstrating significant differences from the White U.S. population. The identification of distinct subtypes, phenotypic correlations, and ethnic variations supports more personalized management strategies for NF1 patients.

## Introduction

Neurofibromatosis type 1 (NF1) is a multisystemic autosomal dominant genetic disorder primarily marked by loss-of-function mutations in the *NF1* gene.^1^ The main clinical manifestations of NF1 involve the development of various nervous system tumors, including cutaneous neurofibromas (cNFs), plexiform neurofibromas (pNFs), and spinal neurofibromas (sNFs). Other notable clinical features of NF1 include café-au-lait macules, skin fold freckles, Lisch nodules, skeletal abnormalities, and the development of malignant peripheral nerve sheath tumors (MPNST) and optic gliomas.^2^, ^3^, ^4^ However, the prevalence and severity of these clinical features have been described mainly in U.S. and European populations,^5^^,^^6^ with only several reports in small Asian cohorts.^7^^,^^8^

Recent data indicate that the estimated global prevalence of NF1 is approximately 1 in 3100 individuals.^9^, ^10^, ^11^ However, significant variations exist across countries, ranging from 1 in 960 in Israel to 1 in 5735 in Italy.^9^^,^^12^^,^^13^ China currently lacks comprehensive studies on the NF1 clinical features within its population. A rough estimate suggests that the number of patients with NF1 in China exceeds 4 times that of the United States,^14^ making it valuable to characterize NF1 phenotypes within the Chinese population.

To address this knowledge gap, we collected clinical phenotype data from 1313 patients with Chinese NF1. We conducted a comprehensive analysis of the prevalence and severity of phenotypic manifestations in this population and compared these with a White U.S.-based NF1 population. We also analyzed how NF1 phenotypes co-associated with each other and explored potential subtypes.

## Method

### Study design and survey distribution

This observational, cross-sectional, multicenter study included 1313 Chinese NF1 participants from November 2022 to November 2023 (Supplementary Fig 1, available via Mendeley at https://data.mendeley.com/datasets/gktzmvrnn5/2). 684 participants (52.1%) were collected from NF1 clinics by physicians, and the remaining 629 (47.9%) were collected through patient-reported surveys administered by the Shanghai Ninth People’s Hospital, Shanghai Jiao Tong University School of Medicine and Xiangya Hospital, Central South University via Wenjuanxing, China’s largest online platform. NF1 data in the U.S. Caucasian population were obtained from the Stanford Dermatology NF1 Genetics Registry Database (May 2021 to December 2023),^15^ with multiple centers offering greater generalizability than single-institution recruitment (Supplementary Methods, available via Mendeley at https://data.mendeley.com/datasets/gktzmvrnn5/2). All participants met the revised NF1 diagnostic criteria.^16^

Surveys were based on prior research and physician input, translated into Chinese from English (Questionnaire, available via Mendeley at https://data.mendeley.com/datasets/gktzmvrnn5/2).^17^ Questionnaires were identical for both the Chinese and U.S. cohorts. The classification of cNFs quantity aligned with previous studies (<10, 11-100, 101-500, and >500).^17^^,^^18^ Our study was approved by IRB. All personal information was de-identified. All results followed STROBE guidelines.^19^

### Data collection

Chinese data were obtained through physician evaluation and a self-reported questionnaire, collaborated with the Neurofibromatosis Shenzhen Care Center partially.^14^ Over 90% of Chinese are Han ethnic. U.S. data were collected through self-reported surveys from the Stanford NF Genetics Registry, which matched NF1 diagnositc criteria. Only questionnaires meeting validation criteria were enrolled.

### Phenotype clustering

Unsupervised k-means clustering identified NF1 subtypes in China,^20^ incorporating the key NF1 diagnostic criteria. The optimal clustering parameter k = 4 was determined using the elbow plot (Supplementary Fig 2, available via Mendeley at https://data.mendeley.com/datasets/gktzmvrnn5/2).

Continuous variables were presented as median, while categorical variables as frequencies and percentages. We performed Spearman correlation analysis for each phenotype pair with Bonferroni correction. To examine the relationship between freckles, café-au-lait macules, and the severity (or presence) of cNFs with other phenotypes, we used logistic regression models adjusted for gender and age. Comparisons with the U.S. cohort used t-test or ANOVA for continuous variables and chi-square test or Fisher's test for categorical variables. *P*-values less than 0.05 were considered statistically significant. Analyses were conducted using R 4.1.2.

## Result

### Population and clinical characteristics

The final study cohort included 1313 Chinese patients, of whom 57.3% were female. The median age at enrollment of the cohort was 19.0 years (IQR: 7.0, 35.0 years), with 627 individuals under age 18 (median age of children 7 years), 435 individuals between 18 and 39 (median age of subset 29 years), and 251 individuals 40 and above (median age of subset 43 years, Table I). 45.8% of participants had prior genetic testing; of these patients, over 90% in each age group reported positive results. Currently, genetic testing was highest (62.8%) among individuals under 18 years old compared to those aged 18-39 (34.7%) and over 40 (22.7%) separately (Table I).

**Table I:** Clinical characteristics of the Chinese NF1 cohort overall and by age group

	All (*n* = 1313)	<18 y (*n* = 627)	18-39 y (*n* = 435)	≥40 y (*n* = 251)
Gender				
Male	560 (42.7%)	304 (48.5%)	159 (36.6%)	97 (38.6%)
Female	753 (57.3%)	323 (51.5%)	276 (63.4%)	154 (61.4%)
Median age (Q1, Q3)	19.0 (7.0, 35.0)	7.0 (4.0, 11.0)	29.0 (24.0, 33.0)	43.0 (41.0, 50.0)
Age diagnosed				
Under 5 y	193 (14.7%)	181 (28.9%)	6 (1.4%)	6 (2.4%)
5-9 y	253 (19.3%)	246 (39.2%)	4 (0.9%)	3 (1.2%)
10-20 y	282 (21.5%)	200 (31.9%)	67 (15.4%)	15 (6.0%)
Older than 20 y	559 (42.6%)	0 (0.0%)	343 (78.9%)	216 (86.1%)
Genetic test	602 (45.8%)	394 (62.8%)	151 (34.7%)	57 (22.7%)
Genetic result				
Yes	547 (90.9%)	357 (90.6%)	137 (90.7%)	53 (93.0%)
pNF	578 (44.0%)	283 (45.1%)	182 (41.8%)	113 (45.0%)
Lisch nodules	69 (5.3%)	22 (3.5%)	19 (4.4%)	28 (11.2%)
Optic gliomas	20 (1.5%)	9 (1.4%)	5 (1.1%)	6 (2.4%)
Sphenoid wing dysplasia	37 (2.8%)	17 (2.7%)	10 (2.3%)	10 (4.0%)
Scoliosis	196 (14.9%)	79 (12.6%)	74 (17.0%)	43 (17.1%)
Bowing bones	31 (2.4%)	12 (1.9%)	13 (3.0%)	6 (2.4%)
sNF	90 (6.9%)	33 (5.3%)	37 (8.5%)	20 (8.0%)
sNF with no treatment	56 (62.2%)	23 (69.7%)	21 (56.8%)	12 (60.0%)
Learning difficulties	224 (17.1%)	101 (16.1%)	84 (19.3%)	39 (15.5%)
Both learning disabilities and attention issues	90 (40.2%)	40 (39.6%)	35 (41.7%)	15 (38.5%)
Long-term itching	263 (20.0%)	141 (22.5%)	88 (20.2%)	34 (13.5%)
Pain	249 (19.0%)	94 (15.0%)	109 (25.1%)	46 (18.3%)
Itching scale (0, 10)	4.7 (2.4)	4.5 (2.1)	5.2 (2.7)	4.0 (2.3)
Pain scale (0, 10)	3.4 (2.2)	3.0 (1.8)	3.6 (2.3)	3.5 (2.4)
Pain location				
Skin	27 (29.7%)	5 (17.9%)	16 (39.0%)	6 (27.3%)
Muscle	21 (23.1%)	7 (25.0%)	6 (14.6%)	8 (36.4%)
cNF				
None	583 (44.4%)	477 (76.1%)	79 (18.2%)	27 (10.8%)
Yes, 1-10	204 (15.5%)	94 (15.0%)	77 (17.7%)	33 (13.1%)
Yes, 11-100	292 (22.2%)	53 (8.5%)	175 (40.2%)	64 (25.5%)
Yes, 101-500	126 (9.6%)	3 (0.5%)	65 (14.9%)	58 (23.1%)
Yes, more than 500	108 (8.2%)	0 (0.0%)	39 (9.0%)	69 (27.5%)

*cNF*, Cutaneous neurofibroma; *pNF*, plexiform neurofibroma; *sNF*, spinal neurofibroma.

44% of Chinese NF1 patients reported pNFs, with no significant differences among age groups. Other phenotypes reported included Lisch nodules (5.3%) and optic gliomas (1.5%), sphenoid wing dysplasia (2.8%), scoliosis (14.9%), and bowing bones (2.4%) (Table I and Supplementary Table I, available via Mendeley at https://data.mendeley.com/datasets/gktzmvrnn5/2). Even though only 90 (6.9%) of patients reported having sNFs, approximately 60% had not received any treatment. Furthermore, of the 224 participants who reported learning difficulties, 90 (40.2%) reported learning disabilities and attention issues concurrently. Chronic itching (20%) and pain (19%) were commonly reported, with the highest prevalence of chronic itching in individuals under 18 (22.5%) and pain in those aged 18 to 39 (25.1%) (Table I). Both itch and pain were generally tolerable, with the maximum reported severity scores of 5.2 and 3.6, respectively. A comparison of online surveys and clinician reports revealed differences in the prevalence of certain phenotypes (Supplementary Tables II and III, available via Mendeley at https://data.mendeley.com/datasets/gktzmvrnn5/2).

**Fig 1:**
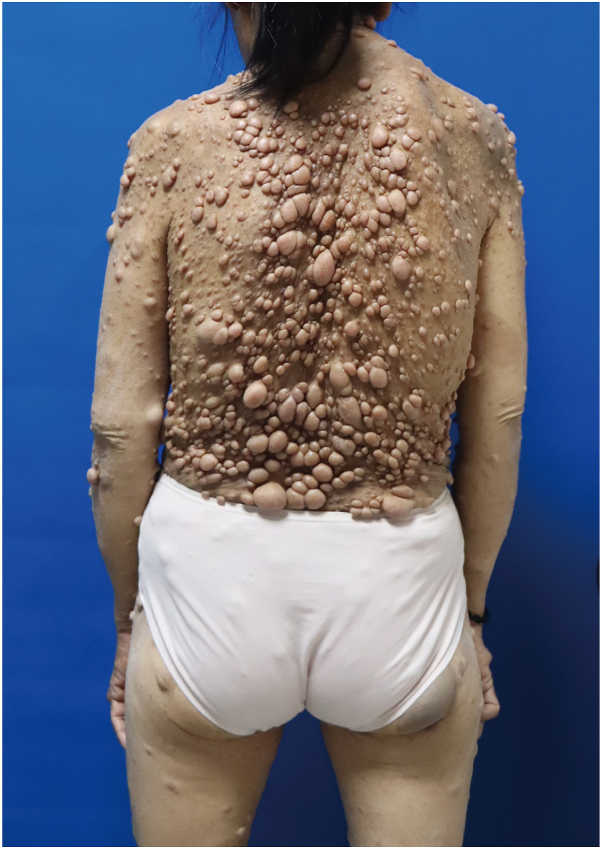
Characteristics of cNFs in patients with Chinese NF1. This figure illustrates the characteristics of cNFs in patients with Chinese NF1.

**Fig 2:**
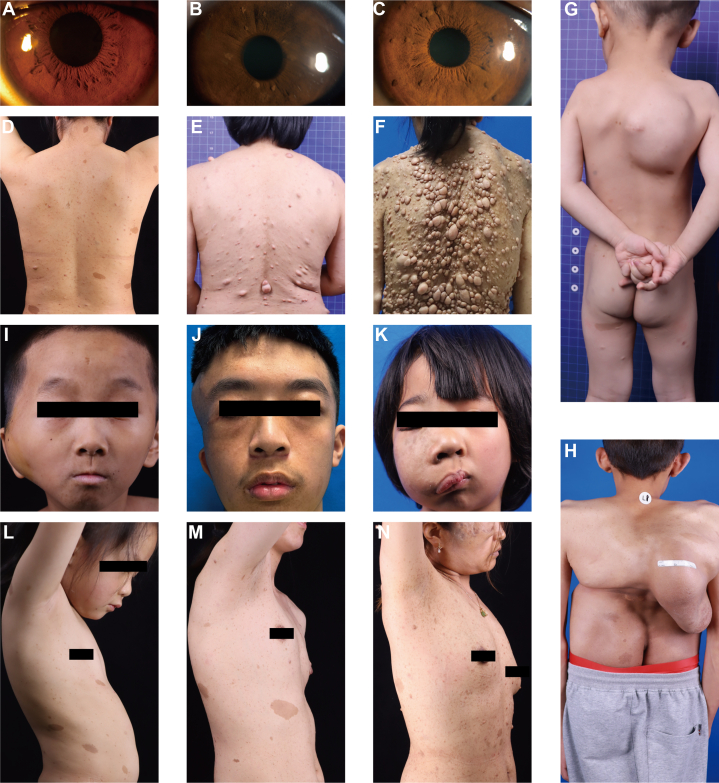
Clinical manifestations of NF1 in Chinese patients. This figure illustrates the diverse clinical features observed in Chinese NF1 patients. The images depict Lisch nodules as absent **(A)**, mild **(B)**, and severe **(C)**; cutaneous neurofibromas as mild **(D)**, moderate **(E)**, and severe **(F)**; scoliosis as mild **(G)** and severe **(H)**; facial plexiform neurofibromas as mild **(I)**, moderate **(J)**, and severe **(K)**; and axillary freckles as mild **(L)**, moderate **(M)**, and severe **(N)**.

The prevalence of cNFs and other manifestations was lower in the NF1 population below 18 years old than in those aged 18 and above, in whom cNFs occurred in over 80% of individuals (Table I). These findings, which are similar to those of prior studies,^21^ suggest younger NF1 population may not yet exhibit the full phenotypic spectrum. Therefore, our primary analysis concentrates on adults ages 18 and above. Classic phenotypic characteristics of patients with Chinese NF1 are presented in Figs 1 and 2.

### Comparison with the U.S. population

For patients with NF1, the phenotype is almost entirely manifested by the age of 40, thus making comparisons of phenotypic variations among those over this age particularly meaningful.^18^ We compared our Chinese NF1 cohort of patients aged 40 and above to a White U.S. cohort (Table II). We observed a significant age difference in NF1 diagnosis between the 2 populations. In the Chinese population, most individuals were diagnosed much later in life, with 86.1% of Chinese participants diagnosed with NF1 at 20 years and older in contrast to 23.3% of the U.S. population (*P* < .001). The Chinese population had a higher proportion of individuals with severe numbers of cNF with 27.5% reporting over 500 cNFs compared with 19.9% of the U.S. Cohort (*P* < .001). The prevalence of MPNSTs was higher in the Chinese population (8.2% vs 4.4%, *P* = .002) as well as fractures, osteoporosis, scoliosis, bowing bones, and pseudarthrosis. In contrast, the prevalence of sNFs was lower in the Chinese population compared to the U.S. population (8.0% vs 27.2%, *P* < .001), while pNFs were similar between the 2 populations (45.0% vs 50.3%).

**Table II:** NF1 traits in Chinese and U.S. study cohorts, and *P*-values for comparisons

	Chinese population (*n* = 251)	American population (*n* = 800)	*P*-value
Age diagnosed			<.001
Under 5 y	6 (2.4%)	269 (33.6%)	
5-9 y	3 (1.2%)	131 (16.4%)	
10-20 y	15 (6.0%)	172 (21.5%)	
Older than 20 y	216 (86.1%)	186 (23.3%)	
Not sure	11 (4.4%)	42 (5.3%)	
cNFs			<.001
None	27 (10.8%)	9 (1.2%)	
Yes, 1-10	33 (13.1%)	94 (12.5%)	
Yes, 11-100	64 (25.5%)	265 (35.2%)	
Yes, 101-500	58 (23.1%)	235 (31.2%)	
Yes, more than 500	69 (27.5%)	150 (19.9%)	
cNFs develop			<.001
Under 8 y	20 (10.3%)	53 (6.6%)	
8-18 y	84 (43.1%)	160 (20.1%)	
19-40 y	82 (42.1%)	418 (52.4%)	
Older than 40 y	8 (4.1%)	107 (13.4%)	
Not sure	1 (0.5%)	60 (7.5%)	
sNF	20 (8.0%)	217 (27.2%)	<.001
pNF	113 (45%)	401 (50.3%)	.152
Lisch nodules	28 (11.2%)	432 (54.1%)	<.001
Optic glioma	6 (2.4%)	101 (12.7%)	<.001
MPNST	12 (8.2%)	35 (4.4%)	.002
Sphenoid wing dysplasia	10 (4.0%)	26 (3.3%)	<.001
Fractures	16 (6.4%)	398 (49.9%)	<.001
Scoliosis	43 (17.1%)	328 (41.1%)	<.001
Bowing bones	6 (2.4%)	112 (13.8%)	<.001
Osteoporosis or osteopenia	15 (6.0%)	189 (23.7%)	<.001
Pseudarthrosis	3 (1.2%)	39 (4.9%)	<.001
Long-term itching	34 (13.5%)	444 (55.7%)	<.001
Pain	46 (18.3%)	515 (64.5%)	<.001
Sweating			<.001
Increase	41 (16.3%)	226 (28.3%)	
Decrease	24 (9.6%)	66 (8.3%)	
Absent	2 (0.8%)	14 (1.8%)	
Normal	184 (73.3%)	492 (61.7%)	
Learning difficulties	39 (15.5%)	439 (55.1%)	<.001
NF1 family history	97 (38.6%)	497 (62.4%)	<.001

*cNF*, Cutaneous neurofibroma; *MPNST*, malignant peripheral nerve sheath tumor; *pNF*, plexiform neurofibroma; *sNF*, spinal neurofibroma.

### Clustering analysis

**Fig 3:**
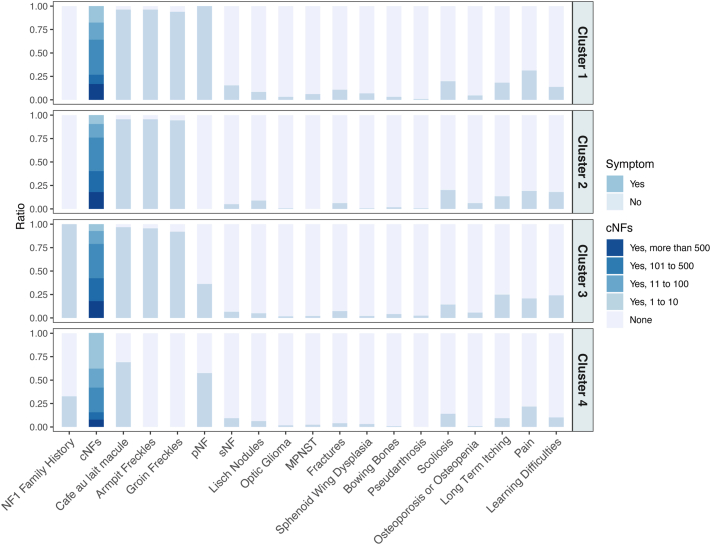
Clinical phenotype clustering of 686 patients with Chinese NF1 aged 18 years and older. K-means clustering analysis revealed 4 distinct clinical subtypes based on the prevalence of clinical features. Each bar represents the proportion of patients with specific symptoms. Cluster 1 (Severe neural subtype, *n* = 132): Characterized by universal pNF presence (100%), elevated rates of sNF (15.2%), MPNST (6.1%), sphenoid wing dysplasia (6.8%), and pain symptoms (31.1%). Cluster 2 (Cutaneous subtype, *n* = 179): Characterized by high prevalence of freckles and cNFs (>100), higher rates of Lisch nodules (8.9%), with low neural involvement (sNF 5.0%, pNF 0%) and bone lesions. Cluster 3 (Hereditary subtype, *n* = 247): Characterized by high familial clustering (100% family history), moderate pNF prevalence (36.0%), and increased rates of itching (25.1%) and learning difficulties (23.9%). Cluster 4 (Mild subtype, *n* = 128): Characterized by low prevalence of freckles (0%), reduced cNFs (62.5%) and café-au-lait macules (68.8%), with moderate pNF prevalence (57.8%). *cNF*, Cutaneous neurofibroma; *MPNST*, malignant peripheral nerve sheath tumor; *NF1*, neurofibromatosis type 1; *pNF*, plexiform neurofibroma; *sNF*, spinal neurofibroma.

To explore how phenotypes clustered within the Chinese population, we performed k-means clustering on persons aged 18 and above based on clinical features, which resulted in 4 distinct clinical clusters (Fig 3, Supplementary Table IV, available via Mendeley at https://data.mendeley.com/datasets/gktzmvrnn5/2). Cluster 1 (33.0 ± 13.1 years) is a severe neural subtype characterized by a high prevalence of pNF (100%), sNF (15.2%), MPNST (6.1%), sphenoid wing dysplasia (6.8%), and pain (31.1%). Cluster 2 (34.8 ± 9.9 years) represents the cutaneous subtype, and features a high prevalence of freckles and high numbers of cNFs (>100). Moreover, this subtype exhibits a relatively higher prevalence of Lisch nodules (8.9%), and low proportions of sNF (5.0%), pNF (0%), and bone lesions. Cluster 3 (34.8 ± 9.6 years) is defined as a hereditary cohort. This cluster is characterized by a high proportion of individuals with a family history of NF1 (100%), a relatively low prevalence of pNF (36.0%), and a higher proportion of itching (25.1%) and learning difficulties (23.9%). Cluster 4 (38.7 ± 12.6 years) is a mild cohort, with a low prevalence of freckles (0%), a relatively lower prevalence of cNFs (62.5%) and café-au-lait macules (68.8%), and a moderate prevalence of pNF (57.8%). Furthermore, the features of each cluster remained similar in the population aged 40 and above (Supplementary Fig 3, available via Mendeley at https://data.mendeley.com/datasets/gktzmvrnn5/2). Clusters 1 and 4 of Chinese NF1 population align more closely with the 'severe neural' and 'mild' subtypes within the 6 clusters of the U.S. cohort.

### Correlation among phenotypic traits

**Fig 4:**
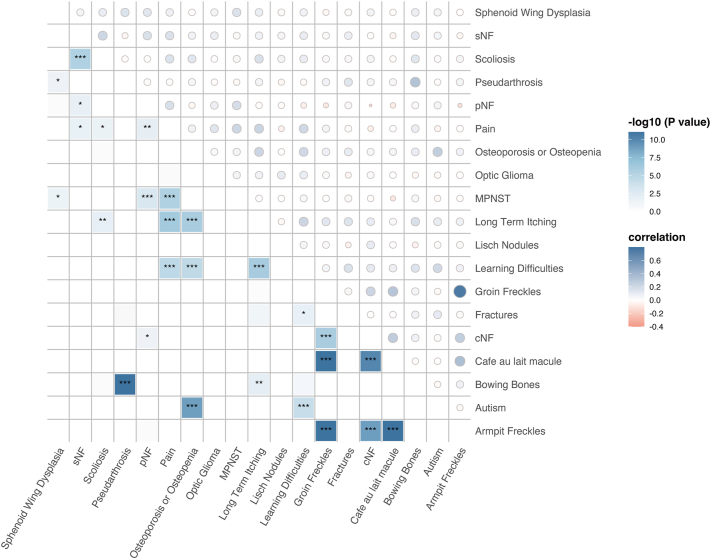
Association of clinical traits in 686 patients with Chinese NF1 aged 18 years and older. This figure illustrates the associations between various clinical traits in patients with NF1 aged over 18 years. The upper right half of the figure displays correlation coefficients, represented by the size and color intensity of the circles, with blue indicating positive correlations and red indicating negative correlations. The lower left half of the figure shows the significance of these correlations after correction, with the color intensity corresponding to the -log10 of the *P*-values. Significance levels are denoted by asterisks: ∗*P* < .05, ∗∗*P* < .01, and ∗∗∗*P* < .001.

We observed a number of correlations across cutaneous and skeletal phenotypes (Fig 4, Supplementary Table V, available via Mendeley at https://data.mendeley.com/datasets/gktzmvrnn5/2). Both skin and skeletal manifestations tended to correlate together in patients. Freckles correlated with café-au-lait macules (armpit freckles: r = 0.349, *P* < .001; groin freckles: r = 0.312, *P* < .001) and cNFs (armpit freckles: r = 0.262, *P* < .001; groin freckles: r = 0.224, *P* < .001), while café-au-lait macules correlated with cNFs (r = 0.275, *P* < .001). Skeletal lesions showed similar correlations, with pseudarthrosis associated with both bowing bones (r = 0.322, *P* < .001) and sphenoid wing dysplasia (r = 0.147, *P* < .05). We also identified positive correlations between sNF and symptoms such as pain (r = 0.154, *P* < .05) and scoliosis (r = 0.218, *P* < .001). Additionally, we observed associations between MPNST and pNF (r = 0.178, *P* < .001), sphenoid wing dysplasia (r = 0.150, *P* < .05), and pain (r = 0.218, *P* < .001). Notably, while most phenotypes exhibited primarily positive correlations, a weak negative correlation was identified between the presence of cNFs and pNF (r = −0.147, *P* < .05). The correlation trends in the subgroup aged ≥40 were consistent with those in the overall adult cohort (Supplementary Fig 4 and Table VI, available via Mendeley at https://data.mendeley.com/datasets/gktzmvrnn5/2).

### Cutaneous findings related to other phenotypes

To further explore the relationship between cutaneous and systemic manifestations, we performed separate logistic regression analyses in the adult Chinese cohort 18 and older (Supplementary Tables VII and VIII, available via Mendeley at https://data.mendeley.com/datasets/gktzmvrnn5/2), as well as in a pediatric cohort below 18 years old (Supplementary Table IX, available via Mendeley at https://data.mendeley.com/datasets/gktzmvrnn5/2). In the adult cohort, the risk of developing pNF was inversely proportional to the number of cNFs and freckles, significantly for cNF numbers over 10 (cNF number: 1-10: OR = 0.67 [0.39-1.14], *P* = .142; cNF number: 11-100: OR = 0.45 [0.28-0.71], *P* = .001; cNF number more than 100: OR = 0.33 [0.20-0.53], *P* < .001; Armpit Freckles: OR = 0.44 [0.28-0.67], *P* < .001; Groin Freckles: OR = 0.47 [0.31-0.70], *P* < .001). Conversely, there was a positive association between increased learning difficulties and increasing numbers of cNFs (cNF number: 1-10: OR = 1.62 [0.68-4.07], *P* = .284; cNF number: 11-100: OR = 2.11 [1.02-4.82], *P* = .056; cNF number: more than 100: OR = 4.74 [2.3-10.84], *P* < .001). Compared to patients without cNFs, those with over 100 cNFs reported more long-term itching (OR = 4.16 [2.05-9.23], *P* < .001).

In the pediatric NF1 cohort, children with cNFs had a higher risk of developing Lisch nodules and bowing bones compared to those without cNFs. The presence of skinfold freckling was associated with elevated risks of developing osteoporosis, scoliosis, itching, and learning difficulties (Supplementary Table IX, available via Mendeley at https://data.mendeley.com/datasets/gktzmvrnn5/2).

## Discussion

This study provides the first comprehensive analysis of clinical phenotypes in a large group of patients with Chinese NF1, with Chinese cohort diagnosed at an older age and with more severe numbers of cNFs, skeletal abnormalities, and malignant nerve sheath tumors. The increased malignancy rate may be influenced by variations in genetic backgrounds among different ethnicities or perhaps by enrichment of a severe phenotype due to underdiagnosis of mild NF1 phenotypes in the Chinese population. A crucial area of future research will be establishing the link between clinical subtypes and genetic variations.

Interestingly, the prevalence of Lisch nodules, sNFs, and optic gliomas was lower in the Chinese cohort than in the White U.S. cohort. We hypothesize that this is primarily due to the absence of standardized medical examinations in China, such as slit lamp exam conducted in the Ophthalmology department. Identifying these symptoms requires specialized diagnostic equipment, posing a considerable challenge for detection in typical outpatient settings. The development of comprehensive guidelines for screening all symptoms carries significant clinical importance in establishing uniformity in diagnosis and treatment.

The higher rate of genetic testing among patients under 18 years old may be attributed to several factors. First, genetic testing has been more widely accessible and affordable in recent years. Second, pediatric patients suspected of having NF1 commonly do not match all clinical diagnostic criteria, making genetic testing a valuable tool for early diagnosis. In addition, higher parental attention to their offspring's health, combined with heightened disease awareness, further makes genetic testing more acceptable.

Differences among individuals reporting over 500 cNFs between the Chinese and U.S. cohorts may be influenced by differences in clinical management. On the one hand, cNFs treatment or surgical removal may be more common in U.S. patients compared with Chinese patients, which affect the exact number of tumors over time.^21^ In addition, differences in quantitative assessment of cNFs may also influence these findings as there is currently no standardized validated tool for objective assessment of cNF burden. Future studies should focus on developing reliable and efficient cNF measurement tools, potentially integrating artificial intelligence and imaging technologies to improve accuracy and reproducibility.

Chinese population reported a lower incidence of itching, possibly influenced by cultural or behavioral factors. While several studies have suggested that the prevalence of itching is lower in Chinese populations compared to North American cohorts,^22^ same observation in our NF1 cohort (13.5% vs 55.7%) appears to be greater. Differences in the itching pathophysiology and racial genetic modifiers are probably involved in these outcomes.^23^^,^^24^ Further research is needed to clarify the underlying mechanisms contributing to this pronounced difference.

Our study also identified 4 phenotype-based clusters of NF1 patients: a mild subtype (cluster 4) and a severe neural subtype (cluster 1), with a greater prevalence of neural tumors including, notably, pNFs, sNFs, optic glioma, and MPNST. These 2 subtypes closely correspond to subtypes identified in previous phenotypic analysis of a U.S. cohort, specifically the “mild” subtype and the “severe neural” subtype.^17^ Within the subtypes predominantly characterized by cNFs, 2 additional clusters (clusters 2 and 3) were identified based on the presence or absence of familial inheritance. In the cluster characterized by familial inheritance (cluster 3), there was a slightly higher prevalence of phenotypes such as neurofibromas and bone lesions than in the cluster lacking familial inheritance (cluster 2). Further genotype-phenotype studies are necessary to gain a deeper understanding of the influence of genetics on phenotype development.

Our study benefited from a large sample size, physician-confirmed clinical diagnosis, and extensive survey validations. Limitations include the use of self-report data for a subset of the cohort and that it was administered through an online questionnaire so response rate could not be calculated. In addition, the enrolled NF1 patients may not fully represent the entire Chinese NF1 population. Third, this study did not address the prevalence of NF1 among ethnic minorities in China and other ethnic groups in the United States. Finally, differences in clinical care and patient education may have led to reduced and delayed diagnosis of clinical phenotypes in the Chinese population and may reflect healthcare system and access differences rather than true biological differences.

Our study conducts a comprehensive overview of the phenotype spectrum of NF1 in Chinese and U.S. populations, we also identified differences in NF1 phenotypes and phenotypic co-associations between these groups. If corroborated, these findings can be used to facilitate precise diagnosis and personalized treatment strategies and improve the consistency of medical education and clinical diagnostic methods across countries. Further investigations are warranted to establish the intricate links between these phenotypes and genotypes. These findings have the potential to drive deeper exploration of NF1, offering crucial insights for both Chinese and global research endeavors.

## Conflicts of interest

Sarin is a scientific advisor for NFlection Therapeutics and co-chairs the Cutaneous Neurofibroma Working Group of the Response Evaluation in Neurofibromatosis and Schwannomatosis (REiNS) international collaboration. Wang, Hu, Mo, Shen, Romo, Vera, Liu, Li, and Li declare that they have no competing interests.
